# Supplementary material for: Identification of photoperiod-induced specific miRNAs in the adrenal glands of Sunite sheep (Ovis aries)
Source: Front Vet Sci. 2022 Jul 22;9:888207. doi: 10.3389/fvets.2022.888207 (PMC9354845; doi:10.3389/fvets.2022.888207)
Supplement: Supplementary file 1 [file Data_Sheet_1.ZIP › Supplementary file/miRDeep2 file.zip!/miRDeep2 file/Novel_Structure/SPLP42A3/Novel_571.pdf]

Provisional ID	: NC_019459.2_5007
Score total	: 1.9
Score for star read(s)	: -1.3
Score for read counts	: 0
Score for mfe	: 1.6
Score for randfold	: 1.6
Score for cons. seed	:
Total read count	: 251
Mature read count	: 251
Loop read count	: 0
Star read count	: 0

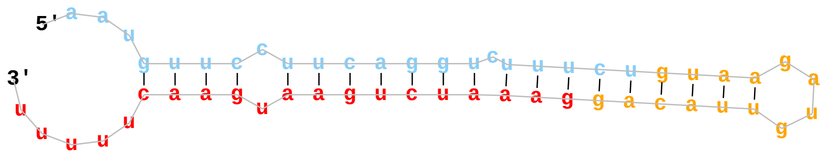

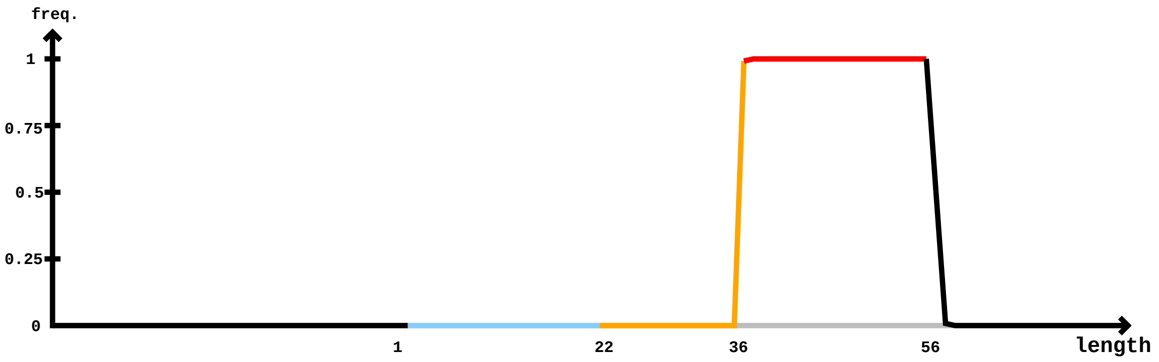

Star Mature

```
5'- cugggaagcccucauacugcuauuggguuggccaaaauguuccuucaggucuuucuguaagauguuacaggaaaucugaaugaacuuuuugaugaauccaguguuaauca   -3'    exp
    ..(((...)))..........(((((((((.(.(((((.((((.(((((((.(((((((((....)))))))))))))))).)))).))))).).)))))))))).......     reads      mm         sample
    ......................................................................gaaaucugaaugaacuuuuu....................     1          0          seq
    ......................................................................Aaaaucugaaugaacuuuuu....................     247        1          seq
    ......................................................................gaaaCcugaaugaacuuuuug...................     1          1          seq
    .......................................................................aaaAcugaaugaacuuuuuga..................     1          1          seq
    .......................................................................aaaucugaaugaacuuuuugC..................     1          1          seq
```
